# *Hyphalus shiyuensis* sp. nov. from Xisha Islands, China (Coleoptera, Limnichidae, Hyphalinae)

**DOI:** 10.3897/zookeys.941.48873

**Published:** 2020-06-16

**Authors:** Zhen-Hua Liu, Qiang Xie, Feng-Long Jia

**Affiliations:** 1 Institute of Entomology, School of Life Sciences, Sun Yat-sen University, Guangzhou, 510275, Guangdong, China Sun Yat-sen University Guangzhou China; 2 Australian National Insect Collection, CSIRO, GPO Box 1700, Canberra, ACT 2601, Australia Australian National Insect Collection Canberra Australia

**Keywords:** *
Hyphalus
*, intertidal zone, new species, Oriental Region, taxonomy

## Abstract

*Hyphalus shiyuensis***sp. nov.** is described from Xisha Islands of China, which represents the ninth species and provides new distribution information for this unique intertidal genus. Brief comparisons between the new species and the known species are given. An updated key to the species of genus *Hyphalus* is provided.

## Introduction

*Hyphalus* Britton, 1971 is a poorly known group of intertidal limnichid beetles and the sole genus in the subfamily Hyphalinae, which has a body shape more similar to Byrrhidae rather than Limnichidae. It was described by Britton (1971) from Heron Island, Australia, and suggested to be a group associated with Limnichidae, Dryopidae, and Elmidae. The reason that Britton included it in Limnichidae as a subfamily was that “I think it undesirable to add the number of families in the Dryopoidea where the family separation is already less marked than is usual” (Britton 1971). Since then seven more species of this genus were described from New Zealand, Japan, and Seychelles (Britton 1973, 1977; Satô 1997; Hernando and Ribera 2000, 2004). All these species are known to live in the intertidal zone, and the larva of *Hyphalus insularis* was reported to be more active in sea water (Britton 1971), which is extremely rare for beetles and even for insects in general.

Recently, we found three specimens collected from Xisha Islands, China, which perfectly fit in the genus *Hyphalus* and are diagnosed as a new species, based on these specimens. We also present an updated key to the species of *Hyphalus*.

## Materials and methods

All the studied specimens of the new species are deposited in the Museum of Biology, Sun Yat-sen University (**SYSU**). Specimens of described species examined in the study are deposited in the Australian National Insect Collection (**ANIC**). Specimens for dissection were prepared in 10% KOH for ca 12 hours, then dissected in glycerol on an open slide under a Leica Sapo stereomicroscope. Habitus was photographed using a Nikon DS-Ri2 mounted on a Nikon SMZ25; layers were captured and aligned in the NIS-Elements software. Individual structures in glycerol were photographed using a Zeiss AxioCam HRc mounted on a Zeiss AX10 microscope with the Axio Vision SE64 software. These images were then aligned in Helicon focus (v7.0.2). SEM images were taken using a Phenom Pro, then also aligned in Helicon focus. All the images were processed and plates were made in Photoshop CC 2019.

The terms used in morphological descriptions follow Lawrence and Ślipiński (2013). Measurements were made as follows: body length from apical edge of clypeus to apex of elytra; body width and elytral width are the maximum width of elytra; pronotal length is the median line from anterior margin to posterior margin; pronotal width is the maximum width of pronotum; elytral length is the length along the elytral suture.

## Systematic classification

### Genus *Hyphalus* Britton, 1971

*Hyphalus* Britton, 1971: 88. Type species: *Hyphalus insularis* Britton, 1971, by original designation.

### Checklist of the described species:

*Hyphalus crowsoni* Hernando & Ribera, 2000: 240.

Distribution: Seychelles, Aldabra Atoll.

*Hyphalus insularis* Britton, 1971: 90.

Distribution: Australia, Queensland, Heron Island.

*Hyphalus kuscheli* Britton, 1977: 82.

Distribution: New Zealand, North Island.

*Hyphalus madli* Hernando & Ribera, 2004: 413.

Distribution: Seychelles, Silhouette Island.

*Hyphalus prolixus* Britton, 1977: 85.

Distribution: New Zealand, North Island.

*Hyphalus taekoae* Satô, 1997: 110.

Distribution: Japan, Ryukyus; China, Taiwan.

*Hyphalus ultimus* Britton, 1977: 85.

Distribution: New Zealand, North Island.

*Hyphalus wisei* Britton, 1973: 121.

Distribution: New Zealand, North Island.

#### Hyphalus shiyuensis sp. nov.

Taxon classificationAnimaliaColeopteraLimnichidae

17C22EC8-05B1-5718-A67B-5D415CEE3A1D

http://zoobank.org/B0F44404-362F-4045-A42C-177A595BE010

Figures 1–9 , 10–13 , 14

##### Material examined.

***Holotype***: male, China, Hainan Province, Xisha, Shiyu Reef, in a small salty pool (中国, 海南, 西沙, 石屿), 16°32'42"N, 111°44'53"E, alt. 0 m, 30.viii.2018, Qiang Xie leg. (SYSU). ***Paratypes***: same data as holotype (2 males, SYSU).

**Figures 1–9.**
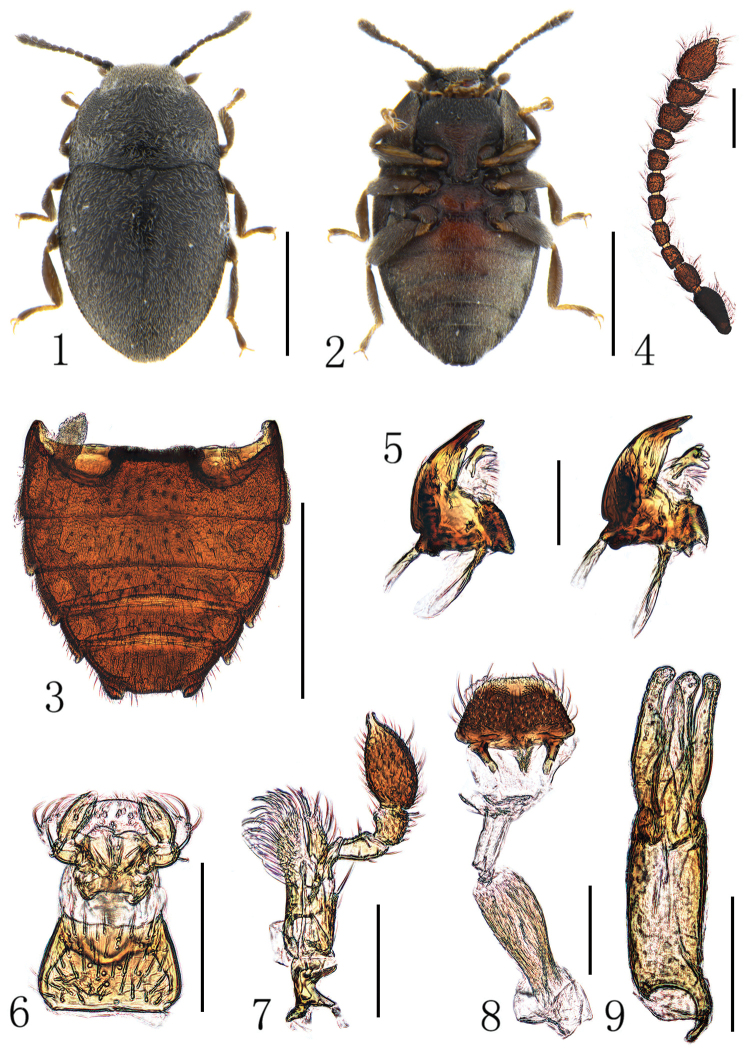
*Hyphalus shiyuensis* sp. nov. **1** habitus, dorsum **2** habitus, venter **3** abdomen, venter **4** antenna **5** Mandibles **6** labium **7** maxilla **8** labrum **9** aedeagus. Scale bars: 0.5 mm for (**1–3**); 0.1 mm for (**4–9**).

##### Additional material examined.

*Hyphalus insularis* Britton, 1971. ***Holotype***: Herron I. Gt. Barrier Reef, Q. 24.xi.1968, beneath rocks below high-water mark. E. Britton, S. Misko (ANIC). ***Paratypes***: same data as holotype (75 specimens, ANIC).

*Hyphalus wisei* Britton, 1973. ***Paratype***: New Zealand Cape Rodney, North I. exposed rock platform opposite Goat I., N. of Leigh 5.xii.68, K.A.J. Wise (dissected for SEM photograph, ANIC). None types: Leigh, NZ G. Kuschel/ *Hyphalus wisei* Britton ♂ (1 male, ANIC).

*Hyphalus kuscheli* Britton, 1977. ***Paratypes***: In rock crevice at H. W. M. Napier Bay 6. III. 1945 J. M. GURR/ Bay of Islands Co. North I. ANIC); In rock crevice at H. W. M. below recent spring H. W. M. Napier Bay 6. III. 1948 J. M. GURR/ Bay of Islands Co. North I. (ANIC).

*Hyphalus prolixus* Britton, 1977. ***Paratypes***: In rock crevice below H. W. M. Otupoho Bay, Moturua, I. 26. III. 1945 J. M. GURR/ Bay of Islands Co. North I. (4 specimens, ANIC).

##### Diagnosis.

The new species can be separated from the New Zealand species by the broadly ovate body shape. Additionally, the median lobe of aedeagus of *H. shiyuensis* sp. nov. is the same length as the parameres (Fig. 9), thus differing from Seychellois *H. crowsoni* and *H. madli*. It can also be distinguished from the Australian *H. insularis* and Japanese *H. taekoae* by the curved basal projection of the phallobase, which is similar to *H. madli* (Hernando and Ribera 2004: fig. 1).

##### Description.

Length 1.10–1.22 mm, width 0.62–0.69 mm. Body compact and nearly ovate (Fig. 1), dorsum black, venter brown to brownish red, slightly convex both dorsally and ventrally. Vestiture of short and dense silver setae (Fig. 2).

Head sub-rectangular, partly retracted in prothorax, not constricted behind eyes; lateral margins slightly curved, posterior margin slightly emarginated; vertex line and occipital incisions absent. Eyes small and very slightly protruding laterally, finely facetted. Antennae closely inserted in front of eyes; insertions concealed by small frontal expansions laterally. Antennae (Fig. 4) 11-segmented with a 3-segmented antennal club, scape elongate and slightly enlarged apically, pedicel smaller and cylindrical, basal two antennomeres of antennal club with small angulate projection at outer apical corner, terminal segment dilated and fusiform; antennomeres with sparse alveolate sensorium on the surfaces (Fig. 11). Frontoclypeal suture present and straight; clypeus rectangular with apical margin very slightly emarginate; labrum (Fig. 8) large and sub-trapezoid, exposed from dorsal side and freely articulated with clypeus. Mandibles (Fig. 5) sub-triangular with broad base and narrow apex, lateral margins curved with three apical teeth, dorsal surface with a lateral tubercle at base; prostheca sclerotized and elongated with several apical setae; mola present. Maxillae (Figs 7, 13) with 4-segmented palps, first palpomere shortest, second palpomere elongate and slightly enlarged apically, third palpomere transverse and short, terminal palpomere enlarged and ovate with pointed apex; galea not narrower than lacinia, apex acute; lacinia with dense long setae along the inner edge. Labium (Fig. 6) small, labial palps 3-segmented, ligula present and broad. Ventral side of head without sub-antennal suture; gular suture widely separated and diverging posteriorly, gula area short. Cervical sclerites present and large.

**Figures 10–13.**
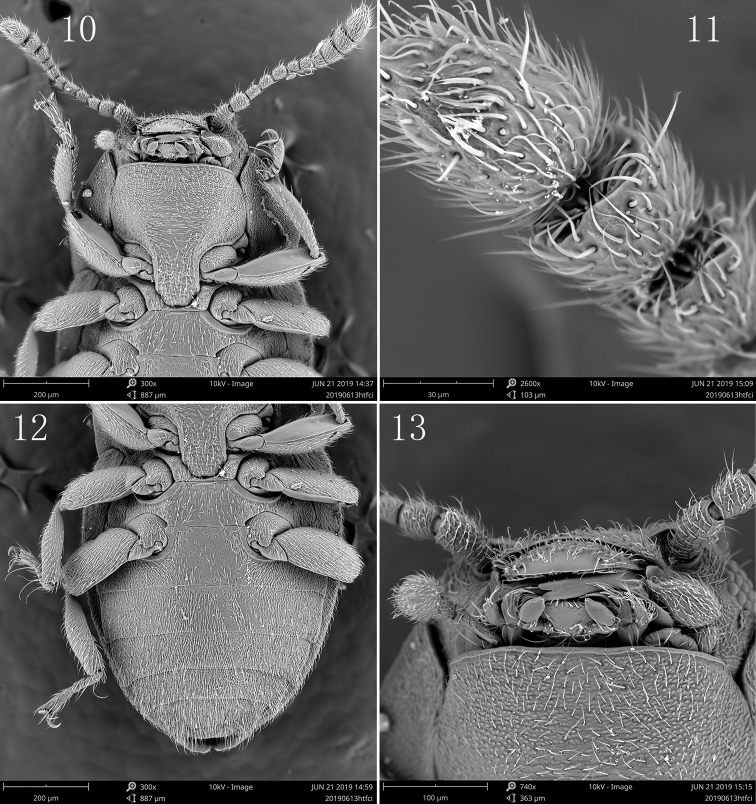
*Hyphalus shiyuensis* sp. nov. SEM images **10** head, prothorax and mesothorax, venter **11** terminal antennomeres **12** pterothorax and abdomen, venter **13** mouthparts.

Pronotum transverse, ca 0.6 times as long as wide, widest just before posterior angles, lateral margins slightly curved, posterior margin bisinuate, anterior angles acute and extending forwardly, posterior angles acute and extending posteriorly; disc convex, with dense and fine punctations; lateral carinae complete, pronotal epipleuron wide. Prosternum with area before procoxae longer than prosternal process, anterior margin broadly curved; prosternal process broad and parallel sided, apex narrowed with truncate apical margin, extending into the cavities on mesoventrite (Fig. 10). Notosternal suture complete. Procoxae slightly transverse with exposed trochantins (Fig. 10), widely separated; procoxal cavities sub-rectangular, externally and internally open.

Scutellum small and triangular. Elytra relatively broad, ca 1.1 times as long as wide, widest at about anterior third, lateral margins crenulate, apex with quadrangular projection that fits into incision of last ventrite. Dorsal surface weakly convex with fine punctations; epipleuron broad at base, extending to the apical projection. Hind wings absent. Mesoventrite short with pair of lateral depressions anteriorly and a large central concavity to receive the prosternal process, mesoventral process broad with posterior margin truncate; metaventrite short and nearly flattened, metanepisternum broad, meso-metaventral junction simple, of straight line; metendosternite with short and very broad strut, lateral arms slender, laminae and anterior tendons absent. Mesocoxae ovate and widely separated, trochantins exposed; mesocoxal cavities laterally open to mesepimeron, distance between cavities larger than width of cavities. Metacoxae ovate and widely separated, only a little wider than length. Legs all with brown enlarged femora, trochanters triangular and yellowish; tibiae flattened and expanded; tarsal formula 4-4-4, first three tarsomeres short and yellowish, last tarsomere elongate and enlarged apically with a pair of falciform claws, all with sparse long hairs underneath.

Abdomen (Figs 3, 12) with five ventrites, gradually narrowed posteriorly, covered with dense short depressed setae which are longer on apex; each segment with pair of small posterolateral projections protruding posteriorly, first three ventrites fused and almost equal in length; intercoxal process of first ventrite broad with anterior margin truncate, fourth ventrite shortest, last ventrite sub-trapezoid with pair of small incisions besides posterolateral projections.

Male genitalia with aedeagus trilobate (Fig. 9), phallobase long and sub-cylindrical with a small basal projection, parameres slender with rounded apex, median lobe bowling-shaped with apex slight enlarged, nearly the same length as parameres.

Female unknown.

##### Habitat.

Living in a small pool filled with sea water on a reef.

##### Etymology.

The new species is named after Shiyu Reef, the type locality. The species name is an adjective.

##### Distribution.

**Figure 14.**
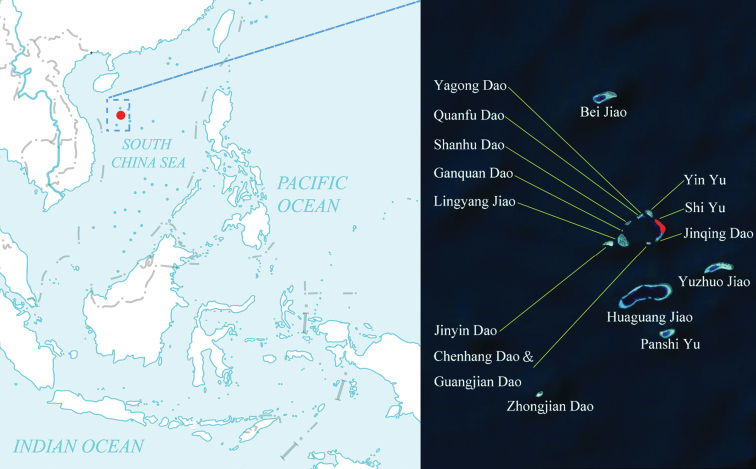
Distribution of *Hyphalus shiyuensis* sp. nov.

Only known from the type locality (Fig. 14).

### A key to the species of genus *Hyphalus* (modified from Britton 1977)

**Table:** 

1	Length more than 1.92 times as long as width	**2**
–	Length less than 1.87 times as long as width	**5**
2	Posterior angles of the pronotum acute	**3**
–	Posterior angles of the pronotum obtuse	**4**
3	Surface of pronotum and elytra bearing obvious tubercles	***H. kuscheli***
–	Surface of pronotum and elytra without obvious tubercles	***H. ultimus***
4	Antennomeres 4 and 5 longer than wide, setae on the base of pronotum in front of the scutellum directed obliquely backwards and outwards	***H. prolixus***
–	Antennomeres 4 and 5 almost of the same length as width, setae on the base of pronotum in front of the scutellum directed obliquely backwards and inwards	***H. wisei***
5	Median lobe of aedeagus shorter than parameres, body length more than 1.8 times width	**6**
–	Median lobe of aedeagus almost the same length as parameres, body length less than 1.8 times width	**8**
6	Antennomeres 8–11 distinctly asymmetric, antennomeres 8–10 each with a prominent denticle on the anterior inner side (Hernando and Ribera 2000: fig. 1)	**7**
–	Antennomeres 8–11 slightly asymmetric, each without prominent denticle on the anterior inner side	***H. insularis***
7	Elytra with tubercles on the whole surface, parameres of aedeagus strongly curved, median lobe narrowed pre-apically (Hernando and Ribera 2004: figs 1, 2)	***H. madli***
–	Elytra with tubercles on the apical region, parameres of aedeagus straight, median lobe not narrowed (Hernando and Ribera 2000: fig. 3)	***H. crowsoni***
8	Denticle on the anterior inner side of antennomere 8 distinctly smaller than that on the antennomere 9 (Satô 1997: fig. 2), phallobase of aedeagus with a broad and less curved projection at base (Satô 1997: fig. 4)	***H. taekoae***
–	Denticle on the anterior inner side of antennomere 8 nearly the same size as that on the antennomere 9 (Fig. 4), phallobase of aedeagus with a slender and strongly curved projection at base (Fig. 9)	***H. shiyuensis***

## Discussion

Among the nine described species of *Hyphalus*, those from New Zealand are distinctly more elongated. After examining the specimens preserved in ANIC, we have found the antennae of *H. insularis*, *H. wisei*, *H. kuscheli* and *H. prolixus* are more or less asymmetrical rather than symmetrical (Hernando and Ribera 2000) and the elytra of those species have apical tubercles similar to those of the new species. It therefore seems likely that all species of *Hyphalus* have asymmetrical antennal clubs and apical tubercles on elytra, although no specimen of *H. ultimus* was examined in this study. Hence, a more detailed study of the morphology of this genus is still needed.

*Hyphalus* is only known from Australia, New Zealand, Seychelles, Japan, and China with nine described species until now. The diversity of this genus, however, might be underestimated given the tiny body size and unique habitats of the species. More careful and comprehensive collection of beetles in the intertidal zones is needed to study the biogeography and dispersal methods of these interesting beetles.

## Supplementary Material

XML Treatment for Hyphalus shiyuensis
